# Associations between *UCP1* -3826A/G, *UCP2* -866G/A, Ala55Val and Ins/Del, and *UCP3* -55C/T Polymorphisms and Susceptibility to Type 2 Diabetes Mellitus: Case-Control Study and Meta-Analysis

**DOI:** 10.1371/journal.pone.0054259

**Published:** 2013-01-24

**Authors:** Bianca M. de Souza, Letícia A. Brondani, Ana P. Bouças, Denise A. Sortica, Caroline K. Kramer, Luís H. Canani, Cristiane B. Leitão, Daisy Crispim

**Affiliations:** 1 Endocrinology Division, Hospital de Clínicas de Porto Alegre, Porto Alegre, Rio Grande do Sul, Brazil; 2 Postgraduate Program in Medical Sciences: Endocrinology, Universidade Federal do Rio Grande do Sul. Porto Alegre, Rio Grande do Sul, Brazil; Central China Normal University, China

## Abstract

**Background:**

Some studies have reported associations between five uncoupling protein (*UCP*) *1–3* polymorphisms and type 2 diabetes mellitus (T2DM). However, other studies have failed to confirm the associations. This paper describes a case-control study and a meta-analysis conducted to attempt to determine whether the following polymorphisms are associated with T2DM: -3826A/G (*UCP1*); -866G/A, Ala55Val and Ins/Del (*UCP2*) and -55C/T (*UCP3*).

**Methods:**

The case-control study enrolled 981 T2DM patients and 534 nondiabetic subjects, all of European ancestry. A literature search was run to identify all studies that investigated associations between *UCP1–3* polymorphisms and T2DM. Pooled odds ratios (OR) were calculated for allele contrast, additive, recessive, dominant and co-dominant inheritance models. Sensitivity analyses were performed after stratification by ethnicity.

**Results:**

In the case-control study the frequencies of the *UCP* polymorphisms did not differ significantly between T2DM and nondiabetic groups (P>0.05). Twenty-three studies were eligible for the meta-analysis. Meta-analysis results showed that the Ala55Val polymorphism was associated with T2DM under a dominant model (OR = 1.27, 95% CI 1.03–1.57); while the -55C/T polymorphism was associated with this disease in almost all genetic models: allele contrast (OR = 1.17, 95% CI 1.02–1.34), additive (OR = 1.32, 95% CI 1.01–1.72) and dominant (OR = 1.18, 95% CI 1.02–1.37). However, after stratification by ethnicity, the *UCP2* 55Val and *UCP3* -55C/T alleles remained associated with T2DM only in Asians (OR = 1.25, 95% CI 1.02–1.51 and OR = 1.22, 95% CI 1.04–1.44, respectively; allele contrast model). No significant association of the -3826A/G, -866G/A and Ins/Del polymorphisms with T2DM was observed.

**Conclusions:**

In our case-control study of people with European ancestry we were not able to demonstrate any association between the *UCP* polymorphisms and T2DM; however, our meta-analysis detected a significant association between the *UCP2* Ala55Val and *UCP3* -55C/T polymorphisms and increased susceptibility for T2DM in Asians.

## Introduction

Diabetes mellitus (DM) has elevated prevalence, morbidity and mortality rates and the social and economic repercussions of its chronic complications compromise both the quality of life and productivity of those affected, making it a serious public health problem. Type 2 DM (T2DM) accounts for 90–95% of DM cases worldwide and usually occurs in obese subjects over 40 years of age. It is characterized by chronic hyperglycaemia caused by a combination of insulin resistance and inadequate compensatory insulin secretion [1].

Type 2 DM is a multifactorial condition and susceptibility to it is determined by the combined effects of multiple genetic and environmental factors [2]. The most likely explanation for the dramatic increase in T2DM prevalence observed over the past two decades is changing patterns of diet and physical activity. However, it is believed that these environmental changes may only lead to T2DM in the presence of a permissive genetic background [2]. Great effort has therefore been exerted in attempts to identify genes associated with T2DM and a number of studies have focused on genes related to energy expenditure, such as those encoding the mitochondrial uncoupling proteins (UCPs) [3], [4], [5], [6].

Uncoupling proteins 1, 2 and 3 are members of an anion-carrier protein family located in the mitochondrial inner membrane [7]. These proteins share structural similarities, but have different tissue expression in mammals [8]. The original UCP, UCP1, is mainly expressed in brown adipose tissue [6], [9]. It was recently shown that under certain pathological conditions, such as hyperglycaemia, UCP1 is also expressed in skeletal muscle, white adipose tissue, retinal cells and pancreatic beta cells [8], [10], [11]. Uncoupling protein 2 is distributed across a wide range of tissue and cell types, whereas UCP3 is mainly restricted to skeletal muscle [5], [9].

Over the last few years, several studies have shown that UCPs reduce metabolic efficiency by dissociating substrate oxidation in mitochondria from ATP synthesis by the mitochondrial respiratory chain. This is thought to be accomplished by promoting net translocation of protons from intermembrane space, across inner mitochondrial membrane, to mitochondrial matrix, thereby dissipating the potential energy available for ATP synthesis and consequently reducing ATP production [12]. This uncoupling effect enables homologue-specific and tissue-specific functions such as thermogenesis (UCP1), regulation of free fatty acid (FFA) metabolism and transport (UCP2 and UCP3), attenuation of production of reactive oxygen species (ROS) by mitochondria (UCP1–3), and regulation of insulin secretion by pancreatic beta cells (UCP2), all of which are mechanisms associated with T2DM pathogenesis [5], [7], [9], [12].

Therefore, the relationship between *UCP loci* and susceptibility to T2DM has been investigated in a number of genetic studies and particular attention has been focused on the -3826A/G (rs1800592) polymorphism in the promoter region of the *UCP1* gene, the -866G/A polymorphism (rs659366) in the promoter region, the Ala55Val (C/T; rs660339) polymorphism in exon 4 and the Ins/Del polymorphism, which is an insertion/deletion of 45 bp in the 3′ untranslated region (3′UTR) of exon 8 of the *UCP2* gene, and the -55C/T (rs1800849) polymorphism in the promoter region of the *UCP3* gene. The results of these studies are not uniform. While some of them have demonstrated associations between one or more of these polymorphisms and T2DM or related characteristics such as obesity, others were unable to detect any associations between the polymorphisms and such characteristics (reviewed in [3], [4], [5], [6], [9]).

Considering that T2DM is caused by interactions between a large number of environmental and genetic factors, it is not unexpected that some genetic association studies will fail to confirm associations with the disease even if they actually exist. Indeed, T2DM has been described as a “geneticist's nightmare” and it may be the case that it will be necessary to study huge numbers of patients in order to elucidate the associations between even a single polymorphism and the disease [13]. Therefore, as part of the ongoing effort to examine the hypothesis that *UCP* polymorphisms are associated with T2DM risk, we performed a case-control study of Brazilian Caucasian subjects followed by a meta-analysis of the literature on the subject.

## Materials and Methods

### Case-control study

#### Ethical approval of the research protocol

The information obtained from the study did not influence patients' diagnosis or treatment. The study protocol was approved by Ethic Committee in Research from Hospital de Clínicas de Porto Alegre and all patients and nondiabetic subjects provided informed consent in writing. All clinical investigation has been conducted according to the principles expressed in the Declaration of Helsinki.

#### Type 2 Diabetes Mellitus population

The sample population comprised 981 unrelated T2DM patients participating in a multicenter study that began recruiting patients in Southern Brazil in 2002. That project was designed to study risk factors for DM and its chronic complications. It initially included four centres in teaching hospitals located in the Brazilian state of Rio Grande do Sul, specifically the Grupo Hospitalar Conceição, the Hospital São Vicente de Paula, the Hospital Universitário de Rio Grande, and the Hospital de Clínicas de Porto Alegre. A detailed description of that study can be found elsewhere [14]. Type 2 DM was diagnosed according to the American Diabetes Association criteria [1].

All patients had European ancestry (mostly of Portuguese, Spanish, Italian and German descent). Ethnicity was defined by self-report. A standard questionnaire was used to collect information on age, age at DM diagnosis and drug treatment and all patients underwent physical examination and laboratory tests. They were weighed barefoot, wearing light outdoor clothing and their height was measured. Body mass index (BMI) was calculated as weight (kg)/height (meters^2^). Blood pressure (BP) was measured twice, in the sitting position, after a 5-min rest, and with a 2-min interval between measurements, using a mercury sphygmomanometer (Korotkoff phases I and V). The means of both measurements were used to calculate systolic and diastolic BP. Arterial hypertension was defined as BP ≥140/90 mm Hg, but patients on antihypertensive drugs were defined as hypertense irrespective of BP at the time of assessment.

The characteristics of the T2DM patients analyzed in this study were as follows: mean age was 59.52±10.63 years, mean T2DM duration was 13.33±9.08 years, mean age at T2DM diagnosis was 46.7±11.6 years, mean glycated haemoglobin (GHb) was 7.18±2.05%, and mean BMI was 28.84±5.39 kg/m^2^. Males comprised 47.4% of the sample, and 72.2% of all patients had arterial hypertension.

#### Nondiabetic sample

The control group contained 534 nondiabetic volunteers attending the blood donation facility at the Hospital de Clínicas de Porto Alegre (Porto Alegre, Brazil) (mean age = 44.0±7.8 years; males = 55.0%). None of these people had DM or a family history of the disease. All of them had European ancestry.

#### Laboratory analyses

Serum samples were collected for laboratory testing after 12 hours fasting. Glucose levels were determined using the glucose oxidase method; GHb was assayed using an ion-exchange HPLC procedure (Merck-Hitachi L-9100 analyzer, Merck, Darmstadt, Germany; reference range: 4.7–6.0%); and total plasma cholesterol, HDL-C, and triglycerides were all tested using enzymatic methods. The fraction of LDL cholesterol was calculated using the Friedewald equation. The nondiabetic subjects did not undergo any of these laboratory tests.

#### Genotyping

DNA was extracted from peripheral blood leukocytes using a standardized salting-out procedure. The -866G/A (rs659366) polymorphism in the promoter region of the *UCP2* gene was detected by digesting polymerase chain reaction (PCR) products with the restriction enzyme *Mlu*I (Invitrogen Life Technologies, Inc., San Diego, CA, USA) as previously described [15]. Digestion fragments were resolved on 2% agarose gels containing GelRed™ Nucleic Acid Gel Stain (Biotium Inc., CA, USA) and viewed under ultraviolet light. A DNA sample with a known genotype (identified by sequencing) was used as a positive control to evaluate the completeness of PCR product digestion. The 45 bp Ins/Del polymorphism in the 3′UTR region of exon 8 of the *UCP2* gene was detected by PCR using primers that have been described elsewhere [16]. The primers amplified products of 457 bp (insertion allele) or 412 bp (deletion allele), which were then resolved on 2% agarose gels stained with GelRed™ Nucleic Acid Gel Stain and viewed under ultraviolet light [17]. Genotypes of the -866G/A and Ins/Del polymorphisms were recorded using the ImageMaster System VDS (GE HealthCare, London, UK).

Primers and probes contained in the 40× Human Custom TaqMan Genotyping Assay (Assays-By-Design Service; Life Technologies, Foster City, CA; USA) were used to genotype the Ala55Val (C/T) polymorphism (rs660339) in exon 4 of the *UCP2* gene, the -3826A/G (rs1800592) polymorphism in the promoter region of the *UCP1* gene, and the -55C/T (*rs1800849*) polymorphism in the promoter region of the *UCP3* gene. Reactions were conducted in 96-well plates, in a 5 µl total reaction volume using 2 ng of genomic DNA, TaqMan Genotyping Master Mix 1× (Life Technologies), and Custom TaqMan Genotyping Assay 1× specific for each polymorphism (Life Technologies). The plates were then placed in a real-time PCR thermal cycler (7500 Fast Real Time PCR System; Life Technologies) and heated for 10 minutes at 95°C, followed by 50 cycles of 95°C for 15 seconds and 63°C for 1 minute. Fluorescence data files from each plate were analyzed using automated allele-calling software (System Sequence Detection v.1.4; Life Technologies).

Genotyping success rates were more than 95% for all polymorphisms and the calculated error rate based on PCR duplicates was less than 3%.

#### Statistical analyses for the case-control study

Allele frequencies were determined by gene counting and departures from the Hardy-Weinberg equilibrium (HWE) were verified using goodness-of-fitness χ^2^ tests. Allele and genotype frequencies were compared between groups using the χ^2^ test. Logistic regression analyses were performed to assess independent associations between the *UCP* polymorphisms and T2DM, after adjustment for age and gender. Two-tailed P values<0.05 were considered statistically significant. Statistical analyses were conducted using SPSS version 18.0 (SPSS, Chicago, IL, USA).

### Meta-analysis

#### Search strategy and eligibility criteria

This study was designed and described in accordance with current guidelines [18], [19]. PubMed and Embase were searched systemically to identify all available genetic studies of associations between T2DM and the most-often studied polymorphisms of *UCP* genes (*UCP1* -3826A/G, *UCP2* -866G/A, *UCP2* Ala55Val, *UCP2* Ins/Del and *UCP3* -55C/T) using the following medical subject headings (MeSH): “Diabetes mellitus, type 2” AND (“mitochondrial uncoupling protein” OR “SLC25A27 protein, human” OR “mitochondrial uncoupling protein 2” OR “mitochondrial uncoupling protein 3”) AND (“mutation” OR “frameshift mutation” OR “germ-line mutation” OR “INDEL mutation” OR “mutation, missense” OR “point mutation” OR “codon, nonsense” OR “sequence deletion” OR “polymorphism, genetic” OR “polymorphism, single nucleotide” OR “polymorphism, restriction fragment length”). The search was limited to human and English or Spanish language papers and was completed on August 24, 2012. All of the articles identified were also searched manually to identify any other relevant citations.

Two investigators (D.A.S and A.P.B.) independently reviewed the titles and abstracts of all articles selected in order to evaluate whether the studies were eligible for inclusion in the meta-analysis. Disagreements were resolved by discussion between them and when necessary a third reviewer (D.C.) was consulted. Where abstracts did not provide enough information regarding the inclusion and exclusion criteria, the full text of the article was retrieved for evaluation. We included observational studies (case-control or cross-sectional designs) that compared one or more of the *UCP* polymorphisms in question between a known number of T2DM patients and nondiabetic subjects. Studies were excluded from the analysis if the genotype distributions in control group deviated from those predicted by the HWE, if they did not have sufficient data to estimate an OR with 95% CI or if they did not employ validated genotyping methods. If data were duplicated and had been published more than once, the most complete study was chosen.

#### Data extraction and quality control assessment

Data were independently extracted by two investigators (B.M.S. and L.A.B.) using a standardized abstraction form, and consensus was sought in all extracted items. When consensus could not be reached, differences in data extraction were resolved by a third reviewer (D.C.) and by referencing the original publication. The information extracted from each individual study was as follows: name of first author, publication year, ethnicity and number of subjects in case and control groups, age, gender, BMI, genotype and allele frequencies in case and control subjects and OR (95% CI).

Two investigators (B.M.S and L.A.B.) independently assessed the quality of each eligible study using the Newcastle-Ottawa Scale (NOS) for assessing quality of case-control studies in meta-analysis [20]. The NOS contains eight items, categorized into three dimensions including selection, comparability, and exposure. For each item a series of response options is provided. A star system is used to allow a semi-quantitative assessment of study quality, such that the highest quality studies are awarded a maximum of one star for each item, with the exception of the item related to comparability, which allows two stars to be assigned. The total NOS score therefore ranges from zero to nine stars [21].

#### Statistical analysis for meta-analyses

Control subjects' genotype distributions were tested for conformity with HWE using a goodness-of-fitness χ^2^ test. Gene-disease associations were measured using OR (95% CI) estimation based on the following genetic inheritance models: (1) allele contrast; (2) additive model; (3) recessive model; (4) dominant model and (4) co-dominant model [22], [23].

Heterogeneity was tested using a χ^2^-based Cochran's Q statistic and inconsistency was assessed with the I^2^ metric. Heterogeneity was considered statistically significant at P<0.10 for the Q statistic and I^2^>50% for the I^2^ metric statistic. Where significant heterogeneity was detected, the DerSimonian and Laird random effect model (REM) was used to calculate OR (95% CI) for each individual study and for the pooled effect; where heterogeneity was not significant, the fixed effect model (FEM) was used for this calculation [24], [25].

Meta-regression and sensitivity analyses were carried out to identify key studies with a substantial impact on inter-study heterogeneity. The factors investigated by meta-regression were age, gender, BMI and ethnicity. Sensitivity analyses were performed after stratifying the studies by ethnicity given that the *UCP* polymorphisms show different frequencies in different ethnic groups.

Risk of publication bias was assessed using funnel plot graphics, analyzed both visually and with the Begg and Egger test [26]. The significance of the intercept was determined by the *t* test, as proposed by Egger, with P<0.10 considered indicative of statistically significant publication bias. All statistical analyses were performed using Stata 11.0 software (StataCorp, College Station, TX, USA).

## Results

### Case-control study

**Table 1** lists the genotype and allele frequencies of the *UCP1* -3826A/G, *UCP2* -866G/A, *UCP2* Ala55Val, *UCP2* Ins/Del and *UCP3* -55C/T polymorphisms. The genotype frequencies of all polymorphisms were in agreement with those predicted by the HWE in non-diabetic subjects (P>0.05) and did not differ significantly between T2DM and nondiabetic groups (**Table 1**). These results did not change after adjustment for age and gender (**Table 1**). Furthermore, the allele frequencies of these polymorphisms were similar in T2DM patients and nondiabetic subjects (**Table 1**). It is worth mentioning that the frequencies of these five *UCP* polymorphisms also did not differ statistically when assuming dominant, recessive, additive or co-dominant models of inheritance (P>0.05).

**Table 1:** Genotype and allele distributions of *UCP* polymorphisms in type 2 diabetes patients and non-diabetic subjects.

*UCP* Polymorphisms	Cases	Controls	Unadjusted P*	Adjusted OR, 95% CI/P^†^
*UCP1* -3826A/G	*n* = 981	*n* = 534		
A/A	489 (49.9)	263 (49.3)	0.694	1
A/G	370 (37.7)	211 (39.5)		1.018 (0.696–1.489)/0.926
G/G	122 (12.4)	60 (11.2)		0.984 (0.554–1.748)/0.956
A	0.687	0.690	0.510	-
*G*	0.313	0.310		
*UCP2* -866G/A	*n* = 778	*n* = 435		
G/G	272 (35.0)	152 (34.9)	0.950	1
G/A	372 (47.8)	211 (48.5)		1.136 (0.760–1.697)/0.534
A/A	134 (17.2)	72 (16.6)		1.405 (0.807–2.444)/0.229
G	0.589	0.592	0.909	-
A	0.411	0.408		
*UCP2* Ala55Val	*n* = 784	*n* = 453		
Ala/Ala	265 (33.8)	142 (31.3)	0.539	1
Ala/Val	371 (47.3)	229 (50.6)		0.871 (0.578–1.313)/0.510
Val/Val	148 (18.9)	82 (18.1)		1.116 (0.650–1.917)/0.691
Ala	0.575	0.566	0.716	-
Val	0.425	0.434		
***UCP2*** ** Ins/Del**	*n* = 779	*n* = 461		
Del/Del	379 (48.7)	226 (49.0)	0.699	1
Ins/Del	314 (40.3)	191 (41.4)		0.880 (0.598–1.295)/0.516
Ins/Ins	86 (11.0)	44 (9.6)		1.387 (0.734–2.621)/0.313
Del	0.688	0.697	0.642	-
Ins	0.312	0.303		
***UCP3*** ** -55C/T**	*n* = 822	*n* = 351		
C/C	559 (68.0)	239 (68.1)	0.988	1
C/T	231 (28.1)	99 (28.2)		0.841 (0.561–1.260)/0.400
T/T	32 (3.9)	13 (3.7)		0.678 (0.256–1.796)/0.434
C	0.821	0.822	0.983	-
T	0.179	0.178		

Data are presented as number of carriers (%) or proportion of sample. The control group contained non-diabetic subjects and cases were type 2 diabetic patients.

* P values were computed using χ^2^ tests to compare case and control groups.

† P values were computed using logistic regression analysis and are adjusted for age and gender.

### Meta-analysis

#### Literature search and characteristics of eligible studies

**Figure 1:**
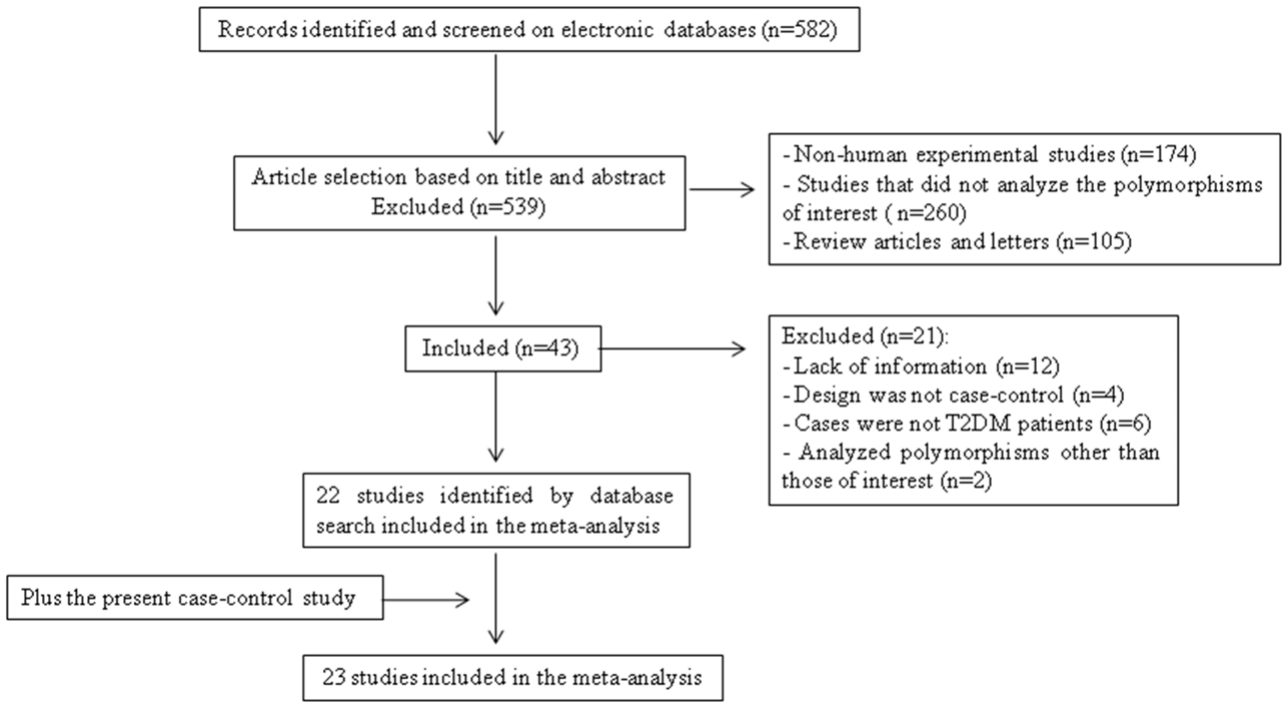
Flowchart illustrating the search strategy used to identify association studies of *UCP1–3* polymorphisms and type 2 diabetes mellitus for the meta-analysis.

**Figure 1** is a flow diagram illustrating the strategy used to identify and select studies for inclusion in the meta-analysis. A total of 582 potentially relevant citations were retrieved by searching the electronic databases and 539 of them were excluded during the review of titles and abstracts. Forty-three articles therefore appeared to be eligible at this point and their full texts were evaluated. However, after reading the full text, another 21 studies were excluded because of missing information or ineligible study designs or because they genotyped other *UCP* polymorphisms, but not the ones of interest here (**Figure 1**). A total of 23 articles fulfilled the eligibility criteria and were included in the meta-analyses: 22 that had been identified through the database searches [15], [27], [28], [29], [30], [31], [32], [33], [34], [35], [36], [37], [38], [39], [40], [41], [42], [43], [44], [45], [46] in addition to the case-control study we describe above, which was also included in the analysis.

**Table S1** summarizes the characteristics of the 23 studies included in the meta-analysis. Seven of these studies analyzed the *UCP1* -3826A/G polymorphism (3071 cases/2561 controls), 12 analyzed the *UCP2* -866G/A polymorphism (4487/4229), 5 analyzed the *UCP2* Ala55Val polymorphism (2112/1841), 3 analyzed the *UCP2* Ins/Del polymorphism (1010/699), and 8 investigated the *UCP3* -55C/T polymorphism (3370/3690). **Table S2** lists the genotype and allele distributions and OR (95% CI) for the *UCP* polymorphisms in case and control samples from the different studies reviewed.

**Table S3** shows the quality of each individual study, assessed using the NOS scale. The highest quality studies were awarded nine stars. In general, most studies were considered as having good quality selection, comparability and exposure. None of the studies scored less than six stars and 47.8% of the studies had eight or nine stars.

#### Quantitative synthesis

**Figure 2:**
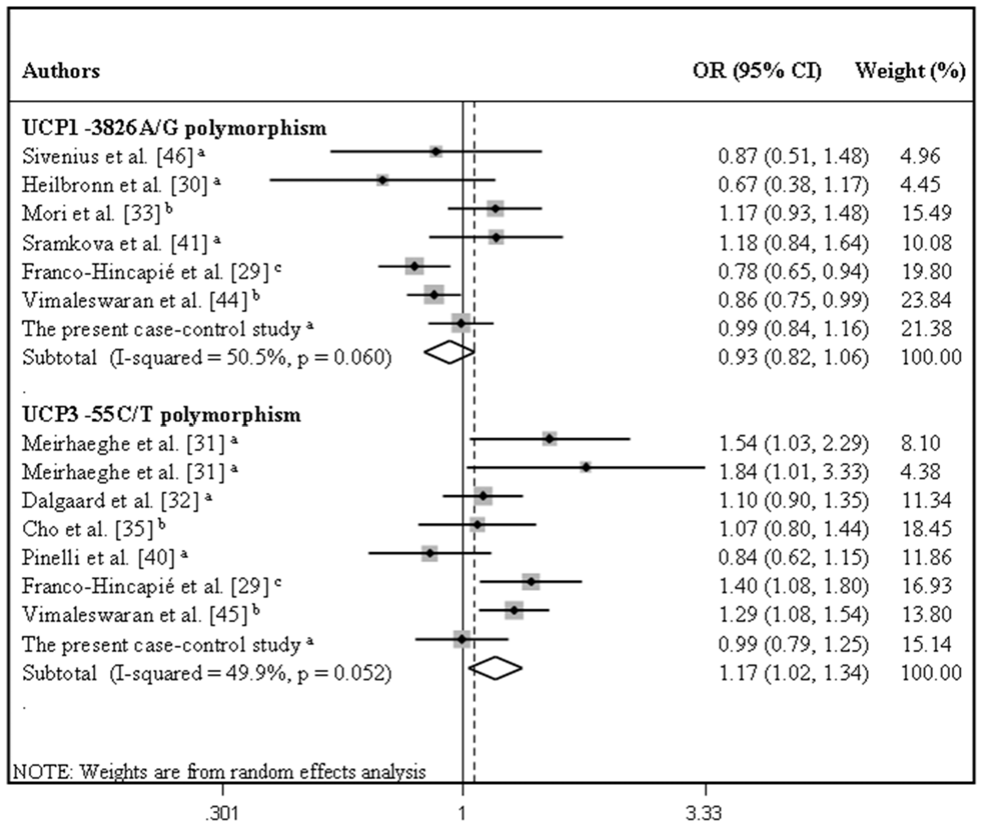
Forest plots showing individual and pooled ORs (95% CI) for the associations between the *UCP1* -3826A/G and *UCP3* -55C/T polymorphisms and type 2 diabetes mellitus under an allele contrast inheritance model. The areas of the squares reflect the weight of each individual study and the diamonds illustrate the random-effects summary ORs (95% CI). ^a^ European population; ^b^ Asian population; ^c^ Mixed population.

**Figure 3:**
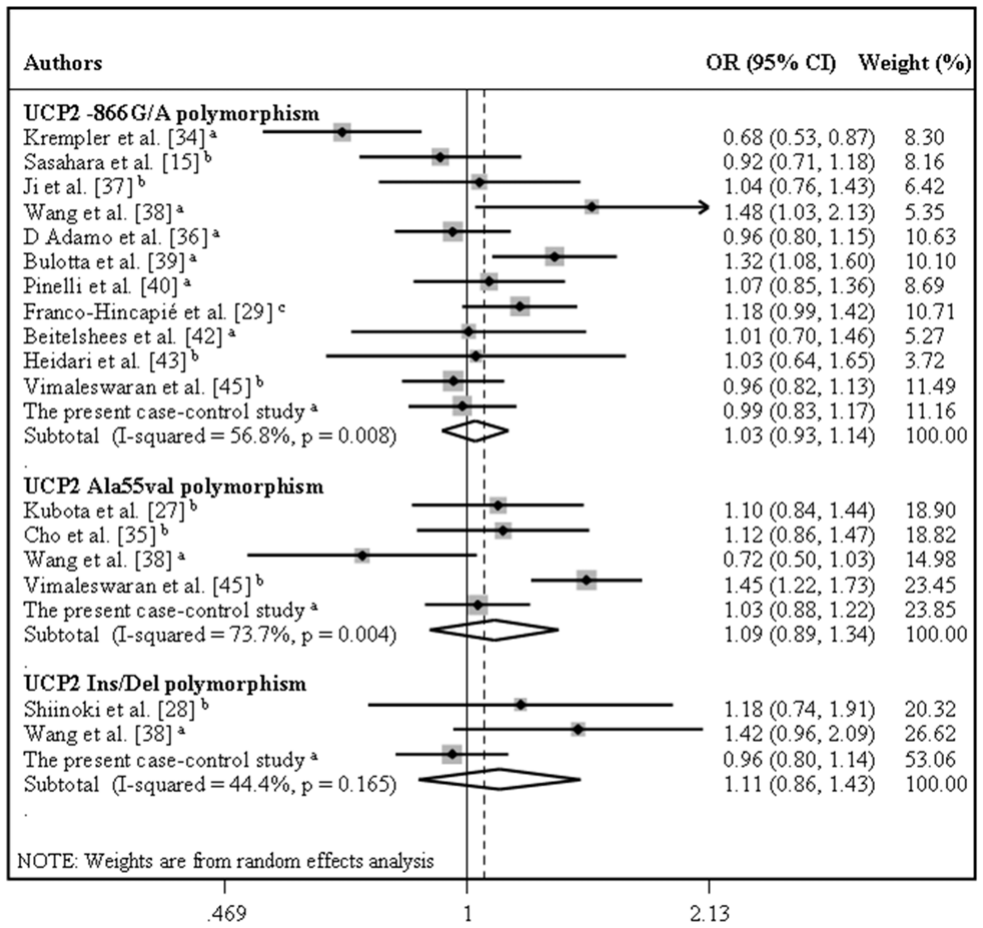
Forest plots showing individual and pooled ORs (95% CI) for the associations between the *UCP2* -866G/A, Ala55Val and Ins/Del polymorphisms and type 2 diabetes mellitus under an allele contrast inheritance model. The areas of the squares reflect the weight of each individual study, and the diamonds illustrate the random-effects summary ORs (95% CI). ^a^ European population; ^b^ Asian population; ^c^ Mixed population.

**Table 2** summarizes the results of the pooled analyses for associations between *UCP* polymorphisms and susceptibility to T2DM. Gene-disease associations were measured for the following genetic inheritance models: allele contrast, additive, recessive, dominant and co-dominant. **Figure 2** illustrates the pooled OR for the associations between T2DM and *UCP1* -3826A/G and *UCP3* -55C/T polymorphisms and **Figure 3** illustrates the pooled OR for the associations between the three *UCP2* polymorphisms and T2DM, both assuming the allele contrast model.

**Table 2:** Pooled measures for associations between the *UCP1* -3826A/G, *UCP2* -866G/A, *UCP2* Ala55Val, *UCP2* Ins/Del and *UCP3* -55C/T polymorphisms and susceptibility to T2DM.

Inheritance model	*n* studies	*n* cases	*n* controls	I^2^ (%)	Pooled OR (95% CI)
***UCP1*** ** -3826A/G**					
Allele contrast overall	7	3,071	2,561	58.0	0.93 (0.82–1.06)
Allele contrast Asian	2	1,130	1,240	81.6	0.99 (0.74–1.33)
Allele contrast European	4	1,391	1,085	10.5	0.98 (0.85–1.14)
Additive	7	3,071	2,561	55.8	0.88 (0.66–1.16)
Recessive	7	3,071	2,561	44.5	0.94 (0.88–1.01)
Dominant	7	3,071	2,561	34.7	0.96 (0.91–1.00)
Co-dominant	7	3,071	2,561	0.0	0.99 (0.94–1.04)
***UCP2*** ** -866G/A**					
Allele contrast overall	12	4,487	4,229	54.8	1.03 (0.93–1.14)
Allele contrast Asian	4	1,159	1,300	0.0	0.97 (0.86–1.09)
Allele contrast European	7	2,788	2,271	70.9	1.03 (0.87–1.22)
Additive	11	4,356	4,111	49.0	1.02 (0.96–1.09)
Recessive	11	4,356	4,111	42.1	1.02 (0.96–1.08)
Dominant	11	4,356	4,111	54.4	1.01 (0.87–1.17)
Co-dominant	11	4,356	4,111	46.2	1.01 (0.97–1.05)
***UCP2*** ** Ala55Val**					
Allele contrast overall	5	2,243	1,959	93.4	1.09 (0.89–1.34)
Allele contrast Asian	3	1,197	1,270	86.5	1.25 (1.02–1.51)
Allele contrast European	2	915	571	97.5	0.90 (0.63–1.27)
Additive	4	1,981	1,723	79.5	1.41 (0.92–2.16)
Recessive	4	1,981	1,723	80.9	1.24 (0.82–1.87)
Dominant	4	1,981	1,723	61.4	1.27 (1.03–1.57)
Co-dominant	4	1,981	1,723	43.2	1.06 (1.00–1.12)
***UCP2*** ** Ins/Del**					
Allele contrast overall	3	1,010	699	44.6	1.01 (0.95–1.08)
Allele contrast European *	2	910	579	68.3	1.12 (0.77–1.63)
Additive	2	879	581	0.0	0.96 (0.84–1.11)
Recessive	2	879	581	0.0	0.96 (0.84–1.09)
Dominant	2	879	581	0.0	1.00 (0.92–1.09)
Co-dominant	2	879	581	0.0	1.02 (0.94–1.11)
***UCP3*** ** -55C/T**					
Allele contrast overall	8	3,370	3,695	63.1	1.17 (1.02–1.34)
Allele contrast Asian	2	986	1,051	76.0	1.22 (1.04–1.44)
Allele contrast European	5	1,839	2,195	59.7	1.12 (0.91–1.38)
Additive	8	3,370	3,695	54.1	1.32 (1.01–1.72)
Recessive	8	3,370	3,695	44.5	1.11 (1.00–1.24)
Dominant	8	3,370	3,695	54.8	1.18 (1.02–1.37)
Co-dominant	8	3,370	3,695	9.5	1.05 (1.00–1.10)

Where significant heterogeneity was detected (I^2^>50%), the DerSimonian and Laird random effect model (REM) was used to calculate OR (95% CI) for each individual study and for the pooled effect; where heterogeneity was not significant, the fixed effect model (FEM) was used for this calculation.

* Stratification analysis was only performed for Europeans for the *UCP2* Ins/Del polymorphism (allele contrast model), since only one study of Asians was identified.

Our results revealed no significant associations between T2DM and *UCP1* -3826A/G, *UCP2* -866G/A or *UCP2* Ins/del polymorphisms, irrespective of whether allele contrast, additive, recessive, dominant or co-dominant models of inheritance were used (**Table 2**). Furthermore, no significant associations were found between these polymorphisms and T2DM when allele contrast models were used after stratification for ethnicity (**Table 2**).

The *UCP2* Ala55Val polymorphism was significantly associated with T2DM risk, but only when assuming a dominant inheritance model (REM OR 1.27, 95% CI 1.03–1.57) (**Table 2**). Furthermore, stratification by ethnicity revealed that, assuming an allele contrast model, the 55Val allele was significantly associated with risk of T2DM in Asians (REM OR 1.25, 95% CI 1.02–1.51), but not in Europeans (REM OR 0.90, 95% CI 0.63–1.27). When assuming the dominant model, the 55Val allele was also associated with risk of T2DM in Asians (REM OR 1.34, 95% CI 1.02–1.76).

The *UCP3* -55C/T polymorphism was significantly associated with T2DM when assuming allele contrast (REM OR = 1.17, 95% CI 1.02–1.34), additive (REM OR = 1.32, 95% CI 1.01–1.72) or dominant (REM OR = 1.18, 95% CI 1.02–1.37) models of inheritance. Additionally, a marginally significant association with T2DM was observed for the recessive (FEM OR = 1.11, 95% CI 1.00–1.24) and co-dominant (FEM OR = 1.05, 95% CI 1.00–1.10) models. Stratification by ethnicity showed that, when assuming an allele contrast model, the *UCP3* -55T allele was associated with T2DM in Asians (REM OR = 1.22, 95% CI 1.04–1.44) but not in Europeans (REM OR = 1.12, 95% CI 0.91–1.38). In the same way, when assuming a dominant model, this polymorphism was associated with T2DM only Asians (REM OR = 1.28, 95% CI 1.06–1.55).

As can be observed in **Table 2**, there was significant heterogeneity between studies investigating the *UCP* polymorphisms when assuming the allele contrast model for the whole sample. Furthermore, significant heterogeneity was also observed between studies when assuming some of the other models of inheritance for these polymorphisms (I^2^>50%; **Table 2**). To investigate this finding in greater depth, sex, age, BMI and ethnicity were used as covariates in the meta-regression analyses performed for the five *UCP* polymorphisms under different inheritance models. None of the covariates used in univariate meta-regression analyses could individually explain the heterogeneity observed (data not shown). Similarly, multivariate meta-regression analyses performed using these covariates also failed to explain the inter-study heterogeneity (data not shown).

Sensitivity analyses were carried out in order to estimate the influence of each individual study on the meta-analysis results obtained when assuming allele contrast inheritance models by repeating the meta-analysis omitting a different study each time. Our results showed that no individual study significantly influenced the inter-study heterogeneity or the pooled ORs for the *UCP2* Ala55Val, *UCP2* Ins/Del and *UCP3* -55C/T polymorphisms (data not shown). In contrast, two studies [34], [39] were largely responsible for the heterogeneity observed in the meta-analysis of *UCP2* -866G/A polymorphism. Heterogeneity was eliminated by exclusion of these two studies (I^2^ = 0% for the allele contrast model), but this did not significantly change the pooled OR (OR = 1.01, 95% CI 0.98–1.05). Similarly, heterogeneity among studies of the *UCP1* -3826A/G polymorphism was effectively reduced (I^2^ = 32.7% for the allele contrast model) by exclusion of one study [33] and the recalculated pooled OR almost reached statistical significance (OR = 0.89, 95% CI 0.79–1.00).

No significant publication bias was detected in any of the inheritance models assessed for any of the *UCP* polymorphisms analyzed (**Figures S1 and S2**), which suggests that our data are statistically robust.

## Discussion

Uncoupling protein 1, UCP2 and UCP3 are regarded as candidate genes for obesity and T2DM because they have been found to decrease mitochondrial membrane potential and mediate proton leak [9], [47]. Mutations reducing the activity or expression of these proteins could theoretically reduce energy expenditure by increasing coupling of oxidative phosphorylation, thereby contributing to the development of obesity and, consequently, T2DM. Moreover, mutations in *UCP2* regulatory regions leading to increased expression could directly cause or exacerbate decreased glucose-stimulated insulin secretion, because of a reduced ATP/ADP ratio in pancreatic beta-cells and could therefore possibly contribute to the development of T2DM [47]. These are the reasons why the roles played by the *UCP1* -3826A/G, *UCP2* -866G/A, *UCP2* Ala55Val, *UCP2* Ins/Del and *UCP3* -55C/T polymorphisms in T2DM risk have been studied extensively, but the results of these association studies remain inconclusive (reviewed in [3], [4], [5], [6], [9], [47]). In an attempt to arrive at a more definitive conclusion about the associations between *UCP* polymorphisms and T2DM, we performed a case-control study of a Brazilian Caucasian population and a meta-analysis of genetic association studies on the subject.

Our case-control study showed that the genotype and allele frequencies of *UCP1* -3826A/G, *UCP2* -866G/A, *UCP2* Ala55Val, *UCP2* Ins/Del and *UCP3* -55C/T polymorphisms were similar in T2DM patients and non-diabetic subjects, suggesting that these polymorphisms are not important risk factors for T2DM in our population. It is also worth mentioning that the frequencies of these polymorphisms in our samples were similar to frequencies observed in other Caucasian populations [27], [28], [30], [31], [34], [35], [40], [41], [42], [45].

Certain factors unrelated to the *UCP* polymorphisms could have interfered with the findings of our case-control study. First, even though only Brazilian Caucasian subjects were studied, both T2DM patients and nondiabetic subjects were recruited from the same hospitals, and the OR obtained was adjusted for age and gender, we cannot rule out the possibility of population stratification bias when analysing our samples. Thus, our results should be interpreted with caution since we did not estimate the ancestral genetic background of our samples using genetic markers, which would be the best way to exclude population stratification bias due to ethnic admixture. Second, we cannot fully exclude the possibility of type II error when analyzing associations between the *UCP* polymorphisms and T2DM. Our statistical power to detect an OR ≥1.4 was >80% for the *UCP1* and *UCP2* polymorphism, but was less than 80% for the *UCP3* -55C/T polymorphism due to the low frequency of the -55T allele. On the other hand, our power was less than 80% for all *UCP* polymorphisms when considering an OR of 1.14 (95% CI 1.12–1.16), which is the mean OR obtained for the ≅20 genetic variants so far consistently associated with risk of T2DM [48]. Moreover, we did not take multiple testing corrections into account when estimating the statistical power. Consequently, our power to detect an association between the analyzed polymorphisms and T2DM could be even smaller. Third, our non-diabetic subjects are approximately 15 years younger than diabetic patients. Although, we tried to minimize this problem adjusting OR by age and sex and selecting a control group with a mean age similar to the mean age at T2DM diagnosis of the diabetic sample, we cannot exclude the possibility that this difference could have influenced our results given that increasing age is an important risk factor for developing T2DM. Notwithstanding these limitations, taking into account that the frequencies of the *UCP* polymorphisms are very similar between T2DM patients and non-diabetic subjects, it seems unlikely that these variants could play an important role in the pathogenesis of T2DM in our population.

Meta-analysis has been considered a powerful tool for pooling the results from different studies that can overcome the problem of small sample sizes as well as inadequate statistical power of genetic association studies of complex traits [49]. Therefore, to further investigate the effects of the *UCP1* -3826A/G, *UCP2* -866G/A, *UCP2* Ala55Val, *UCP2* Ins/Del and *UCP3* -55C/T polymorphisms on T2DM susceptibility, we conducted a meta-analysis of 22 published articles from different populations and also included the results from our case-control study. The results suggest that *UCP1* -3826A/G, *UCP2* -866G/A and *UCP2* Ins/Del polymorphisms are not associated with risk of T2DM in the populations investigated. In contrast, when evaluating allele contrast and dominant models, the *UCP2* 55Val allele was associated with increased risk of T2DM in Asian samples but not in Europeans. Furthermore, the *UCP3* -55T allele also was associated with risk to T2DM in Asian populations under allele contrast and dominant models.

Subjects carrying the Val/Val genotype of the *UCP2* Ala55Val polymorphism appear to have a lower degree of uncoupling of the mitochondrial internal membrane, lower energy expenditure [50], higher exercise energy efficiency [51], higher metabolic rate, increased susceptibility to obesity and T2DM [16], [45], [52], [53] and greater weight loss [54] than subjects with the Ala allele. Notwithstanding, other studies have reported that this polymorphism is not individually associated with basal metabolic rate, metabolic syndrome, BMI, obesity, insulin secretion or T2DM [27], [35], [38], [55], [56], [57], [58]. The T allele of the *UCP3* -55C/T polymorphism has been associated with increased incidence of T2DM [59], higher BMI [60], [61], lower BMI [62], [63], higher HDL cholesterol levels [62], larger waist circumference [64], and high total cholesterol and LDL cholesterol levels and reduced risk of T2DM [31]. However, other studies have not found associations between this polymorphism and metabolic rate, obesity, BMI, insulin secretion or T2DM [55], [57], [65], [66], [67]. The inconsistencies in the results reported by the studies cited here may be at least partially explained by differences in study design, sample size, ethnicity, age, sex and environmental factors and also by synergetic effects with other polymorphisms in the *UCP* genes and in other genes associated with T2DM and/or obesity.

It is well known that functional polymorphisms can influence gene expression, regulating the final quantity of protein in a given tissue, or promote changes in protein activity. The -55C/T polymorphism is located 55 bp upstream of the most commonly used transcription initiation site of skeletal muscle [68]. This polymorphism is potentially interesting because it is located at 6 bp from the TATA box and 4 bp downstream of a putative peroxisome proliferator-activated receptor (PPAR) responsive element and could modify the PPAR responsiveness of the *UCP3* gene [69], [70]. For this reason, it has been suggested that the *UCP3* gene could be one of the PPAR-γ targets involved in the modulation of lipid metabolism and insulin sensitivity [63]. In male Pima Indians, subjects carrying the -55T allele had significantly higher *UCP3* mRNA expression than homozygotes for the -55C allele [71]. Moreover, reduced muscle *UCP3* mRNA levels have been associated with increased BMI in Pima Indians [72]. The increased *UCP3* mRNA expression in -55T allele carriers could explain the association between this polymorphism and lower BMI [62], [63]; however, as already mentioned, other studies have reported an association between this polymorphism and higher BMI [60], [61]. Further studies are still needed to elucidate the functional effects of the *UCP3* -55C/T polymorphism on *UCP3* expression.

The *UCP2* Ala55Val polymorphism causes a conservative amino acid change (alanine/valine) at position 55 of exon 4 and, until now, there had been no evidence that this alteration generates a functional change in the protein [5]. It is therefore possible that this polymorphism may not be a true disease-causing variant, but could simply be reflecting the effects of a functional polymorphism. Taking into account that the Ala55Val polymorphism is tightly linked to the *UCP2* -866G/A polymorphism (|D′| = 0.991) [17], which has a demonstrable effect on *UCP2* gene expression in a number of different tissues (reviewed in [4], [5]), one could hypothesize that the -866G/A polymorphism should be the candidate for the functional polymorphism in the *UCP2* gene. However, our meta-analysis results did not detect any association between the *UCP2* -866G/A polymorphism and T2DM; ruling out a role in T2DM in the populations analyzed. Some other, currently unknown, *UCP2* causative polymorphisms in linkage disequilibrium with the *UCP2* Ala55Val polymorphism must be responsible for the associations reported.

Despite all our efforts, we are aware that certain factors unrelated to the *UCP* polymorphisms analyzed could have interfered with the findings of our meta-analysis. First, meta-analysis is notoriously prone to publication bias, and although we have attempted to trace unpublished observations, we cannot be sure that small negative studies were overlooked. Second, one of the studies identified [57] was not included in the meta-analysis because genotype and allele frequencies for the *UCP2* Ala55Val, *UCP2* -866G/A and *UCP3* -55C/T polymorphisms were not reported for incident diabetic cases and were not provided by the authors. Hsu *et al.* [57] analyzed 14 tag SNPs of the *UCP2*-*UCP3* cluster in an ethnically diverse cohort of postmenopausal women aged >50 years and showed that none of the polymorphisms analyzed remained significantly associated with risk of T2DM after adjustment for multiple testing. Notwithstanding, they reported that a *UCP2*-*UCP3* haplotype (rs591758–rs668517–rs647126–rs1800006) was significantly associated with a greater risk of T2DM especially among overweight Caucasians. This haplotype was not constituted by any of the polymorphisms evaluated in our study. Third, because of the difficulty in getting the full texts of articles published in several languages, we only included studies published in English and Spanish. Fourth, heterogeneity is potentially a significant problem when interpreting the results of any meta-analysis of genetic association studies, and our meta-analysis showed significant inter-study heterogeneity in almost all genetic inheritance models tested. To investigate this issue in greater depth, meta-regression analyses were performed and showed that age, sex, BMI, and ethnicity did not made significant contributions to inter-study heterogeneity. The heterogeneity observed could be due to differences in sample selection, genotyping methods or gene-environment interactions and without detailed information on the metabolic and clinical characteristics of the studies reviewed we could not fully exclude the possibility that the heterogeneity observed might reduce our power to detect true associations. However, sensitivity analyses omitting one study at time did not significantly change the results for associations between the *UCP* polymorphisms and T2DM. Fifth, we also cannot rule out the possibility of type II error when analyzing associations between the *UCP* polymorphisms and T2DM after stratifying by ethnicity. For the whole sample, we had at least 80% power (α = 0.05) to detect even modest ORs (1.17–1.2) for almost all analyzed polymorphisms under the allele contrast model, which is an evidence that our results are robust. However, after stratification by ethnicity, we had 80% power to detect only OR ≥1.3 for all *UCP* polymorphisms (allele contrast model).

It is worth noting that our results are consistent with a previous meta-analysis conducted by Xu *et al.* [73], who analyzed associations between T2DM and *UCP2* Ala55Val, *UCP2* -866G/A and *UCP3* -55C/T polymorphisms in 17 studies, but did not analyze neither the *UCP1* -3826A/G and *UCP2* Ins/Del polymorphisms nor the allele contrast or co-dominant inheritance models. These authors also failed to detect an association between the *UCP2* -866G/A polymorphism and T2DM. In common with our meta-analysis, they also reported that the *UCP3* -55C/T polymorphism was associated with T2DM in Asians when assuming an additive inheritance model (OR = 1.15; 95% CI 1.03–1.28), and in the overall population when assuming dominant (OR = 1.33; 95% CI 1.02–1.73) and recessive (OR = 1.19; 95% CI 1.04–1.36) models. Similarly, they found that the *UCP2* Ala55Val polymorphism was associated with T2DM in Asians for the additive model (OR = 1.23; 95% CI 1.12–1.36), and in the overall population for dominant (OR = 1.42; 95% CI 1.10–1.84) and recessive (OR = 1.39; 95% CI 1.16–1.66) models.

The association between the *UCP2* Ala55Val and *UCP3* -55C/T polymorphisms with T2DM only in Asian populations may be explained in part by the known differences in lifestyle and body weight distributions between Asian and Caucasian populations as well as differences in the genotype frequencies of the analyzed polymorphisms. Luan *et al.* [74] found that the effects of genetic polymorphisms on obesity and related-diseases could be changed by nutritional characteristic of the population. It may be possible that different diet pattern between Caucasian and Asian populations could modulate the effect of *UCP* polymorphism on obesity and diabetes susceptibility.

In conclusion, our results indicate that the *UCP1* -3826A/G, *UCP2* Ins/Del and *UCP2* -866G/A polymorphisms are not important risk factors for T2DM. However, our results strongly suggest that the *UCP2* Ala55Val and *UCP3* -55C/T are associated with susceptibility to T2DM, mainly in the Asian population. The absence of any associations of the five *UCP* polymorphisms and T2DM in our case-control study is in agreement with our meta-analysis results showing important associations of the *UCP2* Ala55Val and *UCP3* -55C/T polymorphisms with T2DM only in Asians. Further additional studies with larger samples are necessary to elucidate the roles possibly played by *UCP* polymorphisms in the pathogenesis of T2DM; particularly studies that analyze the effects of gene-gene and gene-environment interactions.

## Supporting Information

Figure S1 **Funnel plot for contrast allele model for** ***UCP1*** **-3826A/G and** ***UCP3*** **-55C/T polymorphisms.**
(TIF)

Figure S2 **Funnel plot for contrast allele model for** ***UCP2*** **-866G/A, Ala55Val and Ins/Del polymorphisms.**
(TIF)

Table S1 **Characteristics of the eligible studies included in the meta-analysis.**
(DOCX)

Table S2 **Genotype and allele distributions of the** ***UCP1*** **-3826A/G,** ***UCP2*** **-866G/A,** ***UCP2*** **Ala55Val,** ***UCP2*** **Ins/Del and** ***UCP3*** **-55C/T polymorphisms in patients with type 2 diabetes mellitus and nondiabetic subjects.**
(DOC)

Table S3 **Newcastle-Ottawa quality assessment scale for the studies included in the meta-analysis.**
(DOC)
