# Supplementary figures and images for: Associations between UCP1 -3826A/G, UCP2 -866G/A, Ala55Val and Ins/Del, and UCP3 -55C/T Polymorphisms and Susceptibility to Type 2 Diabetes Mellitus: Case-Control Study and Meta-Analysis
Source: PLoS One. 2013 Jan 24;8(1):e54259. doi: 10.1371/journal.pone.0054259 (PMC3554780; doi:10.1371/journal.pone.0054259)

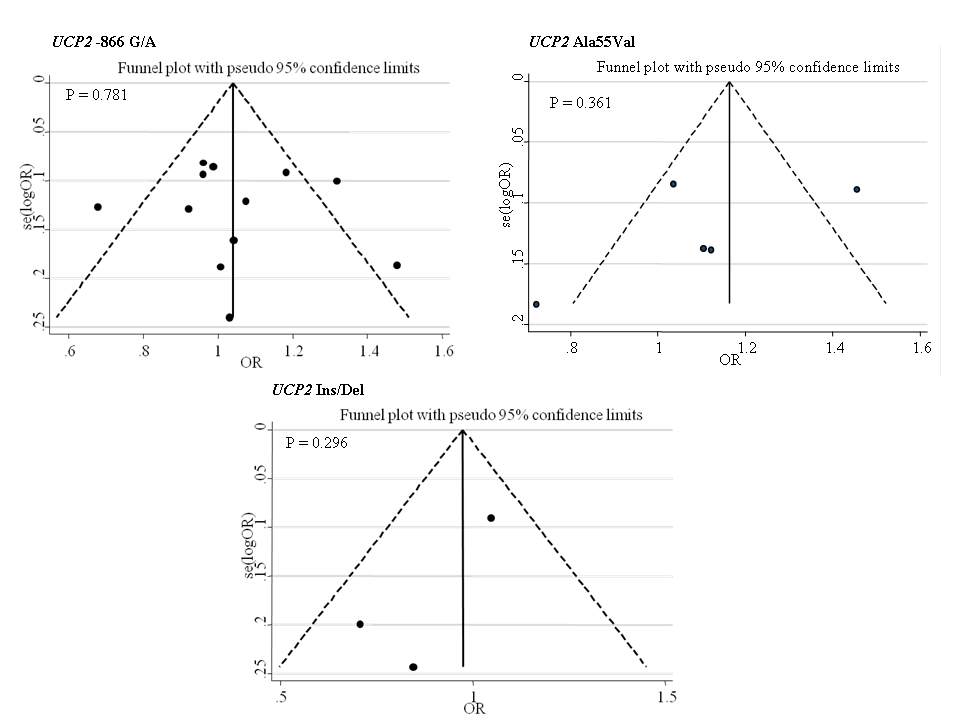

Supplement: Figure S2 — Funnel plot for contrast allele model for UCP2 -866G/A, Ala55Val and Ins/Del polymorphisms. (TIF) [file pone.0054259.s002.tif]
