# Supplementary material for: Associations between UCP1 -3826A/G, UCP2 -866G/A, Ala55Val and Ins/Del, and UCP3 -55C/T Polymorphisms and Susceptibility to Type 2 Diabetes Mellitus: Case-Control Study and Meta-Analysis
Source: PLoS One. 2013 Jan 24;8(1):e54259. doi: 10.1371/journal.pone.0054259 (PMC3554780; doi:10.1371/journal.pone.0054259)
Supplement: Table S1 — Characteristics of the eligible studies included in the meta-analysis. (DOCX) [file pone.0054259.s003.docx]

**Table S1**. Characteristics of the eligible studies included in the meta-analysis.

| **Author [Ref.]** | **Year** | **Ethnicity** | **Polymorphism** | **Age (years)** | **Male (%)** | **BMI (kg/m^2^)** |
| --- | --- | --- | --- | --- | --- | --- |
| Kubota et al. [27] | 1998 | Asian | Ala55Val | 59.8 ± 13.1 | 44.3 | 24.5 ± 4.6 |
| Shiinoki et al. [28] | 1999 | Asian | Ins/Del | 57.0 ± 9.0 | 48.0 | ND |
| Sivenius et al. [46] | 2000 | European | -3826A/G | 60.1 ± 5.8 | 54.0 | 30.5 ± 5.2 |
| Heilbronn et al. [30] | 2000 | European | -3826A/G | ND | ND | ND |
| Meirhaeghe et al. [31] ^a^ | 2000 | European | -55C/T | 56 ± 6.0 | 47.0 | 28.1 ± 4.8 |
| Meirhaeghe et al. [31] ^b^ | 2000 | European | -55C/T | 56.0 ± 7.0 | 47.0 | 31.5 ± 6.6 |
| Dalgaard et al. [32] | 2001 | European | -55C/T | 61.0 ± 11.0 | 58.0 | ND |
| Mori et al. [33] | 2001 | Asian | -3826A/G | 62.9 ± 11.8 | 56.3 | 23.1 ± 3.5 |
| Krempler et al. [34] | 2002 | European | -866G/A | 56.2 ± 12.9 | 48.0 | 36.9 ± 6.0 |
| Cho et al. [35] | 2004 | Asian | Ala55Val; -55C/T | 59.0 ± 10.0 | 48.0 | ND |
| D`Adamo et al. [36] | 2004 | European | -866G/A | 62.0 ± 11.0 | 50.0 | 30.1 ± 6.0 |
| Ji et al. [37] | 2004 | Asian | -866G/A | 60.0 ± 10.0 | 46.2 | 22.4 ± 3.4 |
| Sasahara et al. [15] | 2004 | Asian | -866G/A | 65.5 ± 10.2 | 60.0 | 27.4 ± 4.4 |
| Wang et al. [38] | 2004 | European | -866G/A; Ala55Val; Ins/Del | 62.3 ± 12.3 | 79.0 | 27.6 ± 18.9 |
| Bulotta et al. [39] | 2005 | European | -866G/A | 60.4 ± 8.7 | 51.0 | 30.9 ± 5.6 |
| Pinelli et al. [40] | 2006 | European | -866G/A; -55C/T | 58.0 ± 8.3 | 42.0 | 31.6 ± 6.1 |
| Sramkova et al. [41] | 2007 | European | -3826A/G | 58.8 ± 7.0 | 38.0 | 30.5 ± 5.5 |
| Franco-Hincapié et al. [29] | 2009 | Mixed | -3826A/G; -866G/A; -55C/T | 58.0 ± 10.7 | 35.0 | 27.0 ± 4.6 |
| Beitelshees et al. [42] | 2010 | European | -866G/A | 62.0 ± 12.5 | 65.0 | 29.4 ± 5.9 |
| Heidari et al. [43] | 2010 | Asian | -866G/A | ND | 54.7 | ND |
| Vimaleswaran et al. [44] | 2010 | Asian | -3826A/G | 43.0 ± 13.0 | 43.6 | 26.1 ± 4.2 |
| Vimaleswaran et al. [45] | 2011 | Asian | -866G/A; Ala55Val; -55C/T | 49.0 ± 12.0 | ND | 25.2 ± 4.2 |
| The present case-control study | - | Caucasian-Brazilian | -3826A/G; -866G/A; Ala55Val; Ins/Del; -55C/T | 59.6 ± 10.5 | 42.7 | 28.8 ± 5.4 |

Data are shown as mean ± SD or %; ^a^ case-control study; ^b^ MONICA cohort study; ND, no data.
