# Supplementary material for: Associations between UCP1 -3826A/G, UCP2 -866G/A, Ala55Val and Ins/Del, and UCP3 -55C/T Polymorphisms and Susceptibility to Type 2 Diabetes Mellitus: Case-Control Study and Meta-Analysis
Source: PLoS One. 2013 Jan 24;8(1):e54259. doi: 10.1371/journal.pone.0054259 (PMC3554780; doi:10.1371/journal.pone.0054259)
Supplement: Table S2 — Genotype and allele distributions of the UCP1 -3826A/G, UCP2 -866G/A, UCP2 Ala55Val, UCP2 Ins/Del and UCP3 -55C/T polymorphisms in patients with type 2 diabetes mellitus and nondiabetic subjects. (DOC) [file pone.0054259.s004.doc]

**Table S2.** Genotype and allele distributions of the *UCP1* -3826A/G, *UCP2* -866G/A, *UCP2* Ala55Val, *UCP2* Ins/Del and *UCP3* -55C/T polymorphisms in patients with type 2 diabetes mellitus and nondiabetic subjects

| ***UCP1* -3826A/G** | | | **Cases (n) by total and genotype** | | | | **Controls (n) by total and genotype** | | | | **A allele frequency (%)** | | |
| --- | --- | --- | --- | --- | --- | --- | --- | --- | --- | --- | --- | --- | --- |
| Reference | Year | Ethnicity | Total | A/A | A/G | G/G | Total | A/A | A/G | G/G | Cases | Controls | OR (95% CI)^a^ |
| Sivenius et al. | 2000 | European | 70 | 43 | 26 | 1 | 123 | 81 | 40 | 2 | 80.0 | 82.0 | 0.87 (0.51, 1.48) |
| Heilbronn et al. | 2000 | European | 45 | 22 | 19 | 4 | 99 | 59 | 36 | 4 | 70.0 | 78.0 | 0.67 (0.38, 1.17) |
| Mori et al. [33] | 2001 | Asian | 320 | 83 | 156 | 81 | 250 | 58 | 116 | 76 | 50.3 | 46.4 | 1.17 (0.93, 1.48) |
| Sramkova et al. | 2007 | European | 295 | 157 | 124 | 14 | 120 | 61 | 49 | 10 | 74.2 | 71.3 | 1.18 (0.84, 1.64) |
| Franco-Hincapié et al. | 2009 | Mixed | 550 | 163 | 267 | 120 | 445 | 162 | 211 | 72 | 53.9 | 60.1 | 0.78 (0.65, 0.94) |
| Vimaleswaran et al. | 2010 | Asian | 810 | 292 | 372 | 146 | 990 | 396 | 446 | 148 | 59.0 | 62.5 | 0.86 (0.75, 0.99) |
| The present study | 2012 | European | 981 | 489 | 370 | 122 | 534 | 263 | 211 | 60 | 68.7 | 69.0 | 0.99 (0.84, 1.16) |
| ***UCP2* -866G/A** | | | Cases (n) by total and genotype | | | | Controls (n) by total and genotype | | | | G allele frequency (%) | | |
| Reference | Year | Ethnicity | Total | G/G | G/A | A/A | Total | G/G | G/A | A/A | Cases | Controls | OR (95% CI)^a^ |
| Krempler et al. | 2002 | European | 201 | 65 | 106 | 30 | 391 | 186 | 156 | 49 | 58.7 | 67.5 | 0.68 (0.53, 0.87) |
| Sasahara et al. | 2004 | Asian | 413 | 116 | 205 | 92 | 172 | 50 | 90 | 32 | 52.9 | 55.2 | 0.92 (0.71, 1.18) |
| Ji et al. | 2004 | Asian | 184 | 53 | 94 | 37 | 134 | 37 | 69 | 28 | 54.3 | 53.4 | 1.04 (0.76, 1.43) |
| Wang et al. [38] | 2004 | European | 131 | ND | ND | ND | 118 | ND | ND | ND | 67.0 | 58.0 | 1.48 (1.03, 2.13) |
| D`Adamo et al. | 2004 | European | 483 | 222 | 197 | 64 | 563 | 247 | 266 | 50 | 66.3 | 67.5 | 0.96 (0.80, 1.15) |
| Bulotta et al. [39] | 2005 | European | 746 | 374 | 317 | 55 | 327 | 142 | 144 | 41 | 71.4 | 65.4 | 1.32 (1.08, 1.60) |
| Pinelli et al. [40] | 2006 | European | 342 | 167 | 145 | 30 | 305 | 147 | 124 | 34 | 70.0 | 68.5 | 1.07 (0.85, 1.36) |
| Franco-Hincapié et al. | 2009 | Mixed | 540 | 213 | 251 | 76 | 449 | 144 | 229 | 76 | 62.0 | 58.0 | 1.18 (0.99, 1.42) |
| Beitelshees et al. [42] | 2010 | European | 107 | 37 | 56 | 14 | 341 | 132 | 151 | 58 | 60.7 | 60.9 | 1.01 (0.70, 1.46) |
| Heidari et al. | 2010 | Asian | 75 | 29 | 38 | 8 | 75 | 27 | 41 | 7 | 64.0 | 63.3 | 1.03 (0.64, 1.65) |
| Vimaleswaran et al. | 2011 | Asian | 487 | 185 | 239 | 63 | 919 | 358 | 432 | 129 | 62.5 | 62.5 | 0.96 (0.82, 1.13) |
| The present study | 2012 | European | 778 | 272 | 372 | 134 | 435 | 152 | 211 | 72 | 58.9 | 59.2 | 0.99 (0.83, 1.17) |
| ***UCP2* Ala55Val (C/T)** | | | Cases (n) by total and genotype | | | | Controls (n) by total and genotype | | | | C allele frequency (%) | | |
| Reference | Year | Ethnicity | Total | C/C | C/T | T/T | Total | C/C | C/T | T/T | Cases | Controls | OR (95% CI) ^a^ |
| Kubota et al. | 1998 | Asian | 210 | 60 | 107 | 43 | 218 | 64 | 97 | 57 | 54.0 | 51.6 | 1.10 (0.84, 1.44) |
| Cho et al. | 2004 | Asian | 500 | 158 | 227 | 115 | 133 | 30 | 76 | 27 | 54.3 | 51.1 | 1.12 (0.86, 1.47) |
| Wang et al. [38] | 2004 | European | 131 | ND | ND | ND | 118 | ND | ND | ND | 37.0 | 45.0 | 0.72 (0.50, 1.03) |
| Vimaleswaran et al. | 2011 | Asian | 487 | 264 | 198 | 25 | 919 | 408 | 412 | 99 | 74.5 | 66.8 | 1.45 (1.22, 1.73) |
| The present study | 2012 | European | 784 | 265 | 371 | 148 | 453 | 142 | 229 | 82 | 57.5 | 56.6 | 1.03 (0.88, 1.22) |
| ***UCP2* Ins/Del** | | | Cases (n) by total and genotype | | | | Controls (n) by total and genotype | | | | Del allele frequency (%) | | |
| Reference | Year | Ethnicity | Total | Del/Del | Ins/Del | Ins/Ins | Total | Del/Del | Ins/Del | Ins/Ins | Cases | Controls | OR (95% CI) ^a^ |
| Shiinoki et al. | 1999 | Asian | 100 | 66 | 30 | 3 | 120 | 76 | 38 | 6 | 81.0 | 79.2 | 1.18 (0.74, 1.91) |
| Wang et al. | 2004 | European | 131 | ND | ND | ND | 118 | ND | ND | ND | 75.0 | 68.0 | 1.42 (0.96, 2.09) |
| The present study | 2012 | European | 779 | 379 | 314 | 86 | 461 | 226 | 191 | 44 | 68.8 | 69.7 | 0.96 (0.80, 1.14) |
| ***UCP3* -55C/T** | | | Cases (n) by total and genotype | | | | Controls (n) by total and genotype | | | | C allele frequency (%) | | |
| Reference | Year | Ethnicity | Total | C/C | C/T | T/T | Total | C/C | C/T | T/T | Cases | Controls | OR (95% CI) ^a^ |
| Meirhaeghe et al. ^b^ | 2000 | European | 171 | 116 | 49 | 6 | 124 | 70 | 46 | 8 | 82.2 | 75.0 | 1.54 (1.03, 2.29) |
| Meirhaeghe et al. ^c^ | 2000 | European | 49 | 36 | 13 | 0 | 894 | 542 | 312 | 40 | 86.7 | 78.1 | 1.84 (1.01, 3.33) |
| Dalgaard et al. | 2001 | European | 455 | 252 | 169 | 34 | 521 | 280 | 192 | 49 | 74.0 | 72.2 | 1.10 (0.90, 1.35) |
| Cho et al. | 2004 | Asian | 499 | 251 | 204 | 44 | 132 | 62 | 59 | 11 | 70.7 | 69.3 | 1.07 (0.80, 1.44) |
| Pinelli et al. | 2006 | European | 342 | 240 | 94 | 8 | 305 | 224 | 78 | 3 | 83.9 | 86.2 | 0.84 (0.62, 1.15) |
| Franco-Hincapié et al. | 2009 | Mixed | 545 | 425 | 109 | 11 | 449 | 319 | 112 | 13 | 88.0 | 84.5 | 1.40 (1.08, 1.80) |
| Vimaleswaran et al. | 2011 | Asian | 487 | 278 | 180 | 29 | 919 | 460 | 377 | 82 | 75.6 | 70.6 | 1.29 (1.08, 1.54) |
| The present study | 2012 | European | 822 | 559 | 231 | 32 | 351 | 239 | 99 | 13 | 82.1 | 82.2 | 0.99 (0.79, 1.25) |

^a^ Calculated from the reported genotypes, ^b^ case-control study; ^c^ MONICA cohort study; ND, no data (no genotype data available).
