# Supplementary material for: Associations between UCP1 -3826A/G, UCP2 -866G/A, Ala55Val and Ins/Del, and UCP3 -55C/T Polymorphisms and Susceptibility to Type 2 Diabetes Mellitus: Case-Control Study and Meta-Analysis
Source: PLoS One. 2013 Jan 24;8(1):e54259. doi: 10.1371/journal.pone.0054259 (PMC3554780; doi:10.1371/journal.pone.0054259)
Supplement: Table S3 — Newcastle-Ottawa quality assessment scale for the studies included in the meta-analysis. (DOC) [file pone.0054259.s005.doc]

**Table S3**. Newcastle-Ottawa quality assessment scale for the studies included in the meta-analysis

| **Author [Ref.]** | **Year** | **Selection** | **Comparability** | **Exposure** |
| --- | --- | --- | --- | --- |
| Kubota et al. [27] | 1998 | ** | ** | ** |
| Shiinoki et al. | 1999 | ** | ** | ** |
| Sivenius et al. | 2000 | **** | ** | *** |
| Heilbronn et al. | 2000 | ** | ** | ** |
| Meirhaeghe et al. ^a^ | 2000 | *** | ** | *** |
| Meirhaeghe et al. ^b^ | 2000 | *** | ** | *** |
| Dalgaard et al. | 2001 | *** | * | *** |
| Mori et al. | 2001 | *** | * | *** |
| Krempler et al. | 2002 | **** | ** | *** |
| Cho et al. | 2004 | *** | * | *** |
| D`Adamo et al. | 2004 | *** | ** | *** |
| Ji et al. | 2004 | ** | ** | *** |
| Sasahara et al. | 2004 | *** | ** | ** |
| Wang et al. | 2004 | *** | * | *** |
| Bulotta et al. [39] | 2005 | **** | * | *** |
| Pinelli et al. | 2006 | *** | ** | *** |
| Sramkova et al. | 2007 | *** | * | *** |
| Franco-Hincapié et al. | 2009 | ** | * | *** |
| Beitelshees et al. | 2010 | * | ** | *** |
| Heidari et al. | 2010 | **** | ** | ** |
| Vimaleswaran et al. | 2010 | **** | * | *** |
| Vimaleswaran et al. | 2011 | **** | * | *** |
| The present case-control study | 2012 | **** | * | *** |

^a^ case-control study; ^b^ MONICA cohort study.
